# No serological evidence for Zika virus infection and low specificity for anti-Zika virus ELISA in malaria positive individuals among pregnant women from Madagascar in 2010

**DOI:** 10.1371/journal.pone.0176708

**Published:** 2017-05-16

**Authors:** Norbert Georg Schwarz, Eva Mertens, Doris Winter, Oumou Maiga-Ascofaré, Denise Dekker, Stephanie Jansen, Dennis Tappe, Njary Randriamampionona, Jürgen May, Raphael Rakotozandrindrainy, Jonas Schmidt-Chanasit

**Affiliations:** 1 Research group Infectious disease epidemiology, Bernhard Nocht Institute for Tropical Medicine, Hamburg, Germany; 2 German Center for Infection Research, Hamburg Borstel-Lübeck, Germany; 3 Research group Medicine in the Tropics, Kumasi Centre for Collaborative Research, Kumasi, Ghana; 4 WHO Collaborating Centre for Arbovirus and Haemorrhagic Fever Reference and Research, Bernhard Nocht Institute for Tropical Medicine, Hamburg, Germany; 5 Research group Zoonoses, Bernhard Nocht Institute for Tropical Medicine, Hamburg, Germany; 6 Department of Microbiology and Parasitology, Université d’Antananarivo, Antananarivo, Madagascar; Singapore Immunology Network, SINGAPORE

## Abstract

It was previously reported that a malaria infection may interfere with the specificity of a commercial ELISA test against Zika virus (ZIKV). We analyzed 1,216 plasma samples from healthy, pregnant women collected in two sites in Madagascar in 2010 for ZIKV antibodies using a commercial ELISA and for *Plasmodium* infection by PCR. This screen revealed six putative ZIKV-positive samples by ELISA. These results could not be confirmed by indirect immunofluorescence assays or virus neutralization tests. Four of these six samples were also positive for *P*. *falciparum*. We noted that the frequency of malaria positivity was higher in ZIKV-ELISA positive samples (50% and 100% in the two study sites) than ZIKV-negative samples (17% and 10%, respectively), suggesting that malaria may have led to false ZIKV-ELISA positives.

## Introduction

Zika virus (ZIKV) is a mosquito-borne flavivirus (family *Flaviviridae*), transmitted by *Aedes* mosquitoes. It was first identified in a monkey in the Zika forest of Uganda in 1947 [1]. In the following 60 years, ZIKV was repeatedly shown to circulate in mosquitoes in Africa and Asia, and was also isolated from humans with asymptomatic to mild infection [2, 3].

ZIKV gained global attention after its introduction into Brazil in 2013 [4, 5] and subsequent rapid spread in the Americas starting in May 2015. As of November 2016, autochthonous transmission of ZIKV was reported from 47 countries and territories in South and Latin America [6]; and an association between ZIKV infection and neurological complications including Guillain-Barré syndrome and congenital defects in children born to women infected by ZIKV during pregnancy could be demonstrated [7, 8].

The first larger ZIKV outbreaks were reported from the Pacific region. In a study from a ZIKV-outbreak on Yap Islands (Federate States of Micronesia) in 2007, 49 confirmed and 59 probable cases of symptomatic ZIKV disease were identified and a seroprevalence of 73% anti-ZIKV IgM antibody positives in over three-year olds was observed [9]. In 2013, French Polynesia was hit by a ZIKV epidemic with approximately 28,000 cases corresponding to 11% of the population [10]. During this outbreak, Guillain-Barré syndrome [10] and microcephaly [11] were first shown to be associated with ZIKV infection. Seroprevalence for anti-ZIKV antibodies in French Polynesia before the 2013 outbreak was estimated to be 0.8% based on screening banked blood from 593 blood donors [12].

There are two known ZIKV lineages, one African and one Asian. The reported outbreaks in the Pacific as well as the Americas were caused by the Asian linage, suggesting an Eastward spread from Southeast Asia into the Pacific and the Americas [13].

Although Madagascar was described as potential ecological environment for ZIKV transmission [14], there are no reports from Madagascar on the presence of ZIKV after clinical or serological examinations. However, ZIKV infection may remain unrecognized as it usually presents with only mild disease or asymptomatic infection.

Direct evidence of ZIKV infection by PCR is only possible in the acute phase of infection. Different serological methods are used to detect antibodies against ZIKV in serum including IgG and IgM ELISA, indirect immunofluorescence assays (IIFA) and virus neutralization tests (VNT). All these methods bear risks of cross-reactivity with other flaviviruses, most notable dengue virus (DENV) [15]. VNT have a higher specificity since they directly test the activity of neutralizing antibodies in serum towards live ZIKV.

A previous study demonstrated high specificity of ELISAs for the detection of anti-ZIKV antibodies, when using serum samples from patients with different flavivirus infections [16]. However, recently, false positive results were described with serum samples from malaria patients [17].

In this seroprevalence study we investigated the presence of ZIKV antibodies in archived plasma samples that were collected in Madagascar from pregnant women in 2010, and for which serology for CHIKV, DENV and PCR for malaria had been performed previously in order to assess if ZIKV was circulating on the island at that time. To confirm the Zika-ELISA results, IIFA and VNT were performed.

## Methods

### Plasma sample set

**Fig 1:**
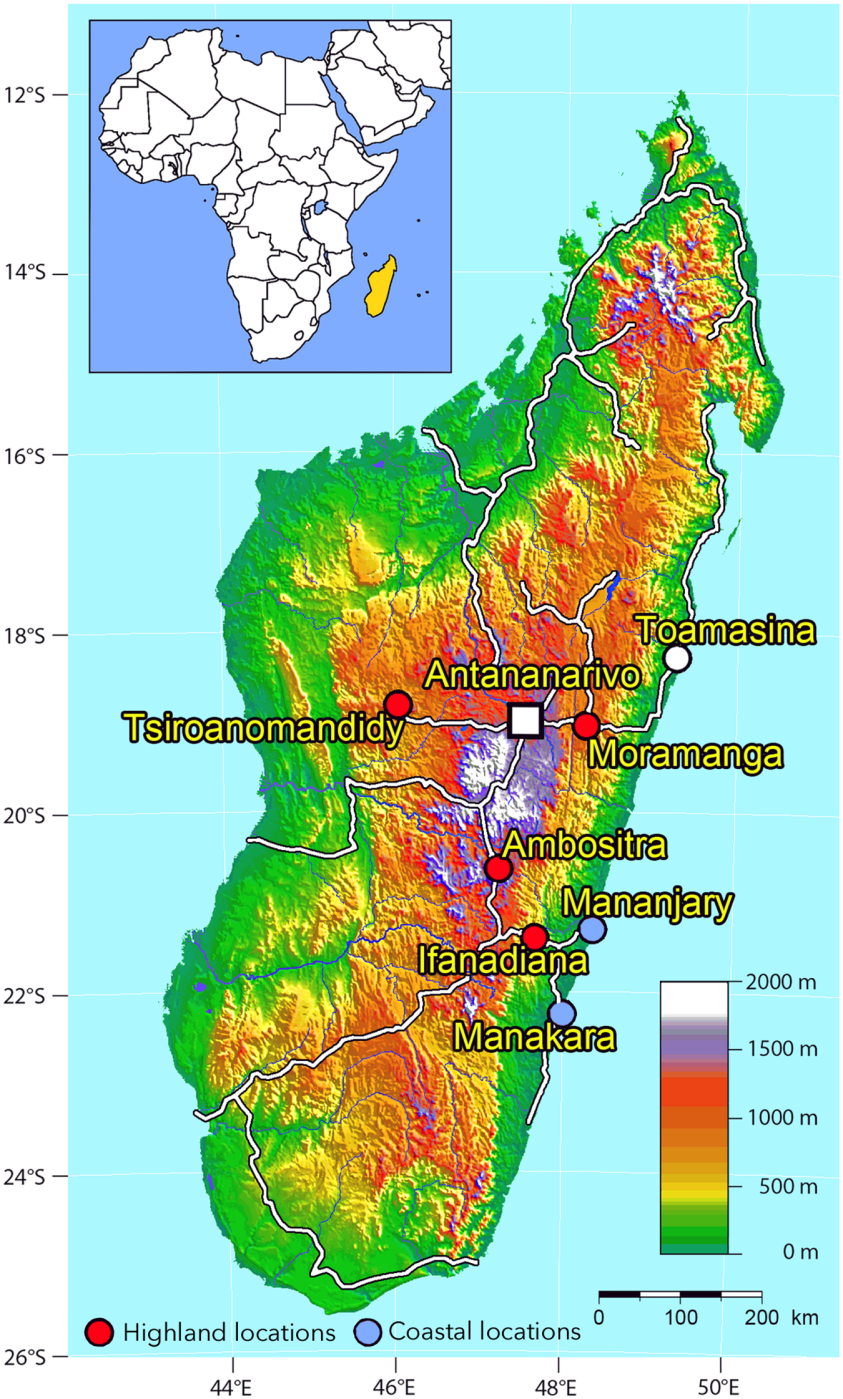
Locations in Madagascar, where the plasma samples investigated in this study were collected in 2010. Locations at seal level (blue dots, n = 433) and in the highlands (red dots, n = 783) were analyzed separately.

Plasma samples stem from cross-sectional surveys that were carried out between May and July 2010 in pregnancy follow-up services in six different locations of Madagascar. Venous EDTA blood samples were collected for a study on malaria parasitemia in pregnant women [18] and for investigation of a previous outbreak of a arboviral infection hat had taken place around 3–4 months before sampling at the Eastern Coast of Madagascar in the surroundings of the cities of Mananjary and Manakara [19]. Supernatant plasma was stored at -20°C for serological analysis. Plasma samples were collected at six different locations aiming for 200 plasma samples from each location (Fig 1). Two of the locations were at the East Coast at sea level (Mananjary and Manakara). Further four locations were highland locations on different elevation levels at 466m (Ifanadiana), 860m (Tsiroanomandidiy), 920m (Moramanga) and 1280m (Ambositra).

### Ethical approval

Blood was collected from all pregnant women presenting for routine pregnancy screenings to the local health centre who gave consent to their participation in the study. All participants provided written informed consent to participate in the study. Ethical clearance for malaria and antiviral antibody testing was obtained from the “Comité d’ethique de la Vice Primature Chargée de la Santé Publique”.

### Enzyme Linked Immunosorbent Assays (ELISA)-testing

For the detection of IgG or IgM antibodies to ZIKV we used an anti-ZIKV ELISA (IgG/IgM) assay (Euroimmun, Lübeck, Germany), which uses the recombinant ZIKV-non-structural protein 1 (NS1) to minimize cross-reactivity to other flaviviruses. The measurement against a manufacturer-provided calibrator led to a ratio of extinction value for the sample to extinction value for the calibration solution. The manufacturer suggests interpreting the resulting ratios by classification into three categories:

Extinction Ratio < 0.8:     Negative
Extinction Ratio ≥ 0.8 - ≤1.1:  Borderline
Extinction Ratio > 1.1:     Positive

### Indirect immunofluorescence assays (IIFA)

IIFA for ZIKV was performed with ZIKV strain MR766-infected Vero E6 cells. In brief, infected Vero cells were spread onto slides, air dried, and fixed in acetone. Plasma samples were serially diluted in phosphate-buffered saline (PBS) starting with an initial dilution of 1:10, added to the cells, and incubated for 90 min at 37°C. After washing with PBS, slides were incubated with fluoresceine isothiocyanate-labeled rabbit anti-human IgG and IgM antibodies at 37°C for 25 min. IgG titers or IgM titers of 1:20 or more were considered positive [20].

### Detection of antibodies against chikungunya virus (CHIKV) and dengue virus (DENV)

Anti-CHIKV-IgG und Anti-DENV-IgG were also detected using immunofluorescence [21] analogous to the procedure described above.

### Viral neutralization tests (VNT)

For the VNT serial twofold dilutions from 1:20 to 1:2,560 were prepared for each plasma, and a volume of 50μL from each dilution was transferred to microtiter plates. A volume of 50μL containing 50 PFU of ZIKV strain MR766 was added to each well except for the controls, which contained phosphate-buffered saline (PBS). The plates were incubated at 37°C for 1h. Then, 100μL of the plasma/virus mixture were transferred to a 96-well plate of Vero cells containing approximately 2x10E5 cells/well in EMEM enriched with 1% fetal bovine serum, 1% penicillin-streptomycin, 1% [200mM] L-glutamine, 1% kanamycin, and 3% amphotericin B and incubated at 37°C in the presence of 5% CO_2_. After six days, the microtiter plates were fixed in formaldehyde, stained with crystal violet, and the presence/absence of cytopathic effect was read under an inverted microscope.

### Sample size considerations for assessing ZIKV circulation in Madagascar in 2010: Sensitivity and specificity of the diagnostic test and certainty of conclusions

As our aim was to assess if ZIKV circulated in the human population of Madagascar in 2010 without having evidence for clinical manifestations or an outbreak of ZIKV, we assumed a low seroprevalence. Sample size considerations were focused on the coastal samples as the probability for the presence of ZIKV was expected to be higher at the coast. We could perform the ELISA on 433 samples from the coast. For the EUROIMMUN ELISA, the specificity, based on samples from 1,015 uninfected individuals, was estimated to be 99.8% [22].

The probability for a single test of a negative sample to be positive is 1-specificity and for a specificity of 99.8%, this would be 0.2%. Using a binomial distribution, we thus get a probability of 58.0% to find at least one (false) positive sample in 433 tests.

$$P\left(x\geq 1\right)=1-P\left(x=0\right)=1-\;\left[\left(\begin{array}{c} 433\\ 0 \end{array}\right)\;0.002^{0}\;0.998^{433}\right]$$

When generalizing this formula to

$$P\left(x\geq k\right)=1-\sum_{k}^{n}\left(\begin{array}{c} n\\ k \end{array}\right)b^{k}a^{n-k}$$

*k* = *minimum number of positives, n* = *sample size, a* = *specificity, b* = *1-specificity*

one can calculate the probability for getting different numbers of false positives (among samples that in reality are all negative). As calculating cumulative probabilities by hand is quite cumbersome we used and online tool for our assumptions (http://stattrek.com/online-calculator/binomial.aspx). For at least two positive samples among 433 tests this probability would be 21.5% for at least three positive samples 5.7% and for at least four positive samples 1.2%. These are the probabilities to falsely claim ZIKV occurrences based on the respective number of positively tested samples under the assumption that the test specificity was indeed 99.8%. Prevalence estimates can be statistically corrected for the imperfection (specificity <1) of a diagnostic test [23].

### Molecular detection of *Plasmodium* species

After DNA extraction from peripheral venous blood-EDTA/8 M urea (v/v) using the QIAamp^®^ DNA Blood mini Kit according to the manufacturer’s protocol (Qiagen), infection with *Plasmodium* parasite was determined by using a real-time PCR methodology. *Plasmodium falciparum* was detected with primers and probes targeting the *cox1* gene and *P*. *ovale*, *P*. *malariae* and *P*. *vivax* with primers and probes targeting the *18S rDNA* [18].

On a LightCycler 480 (Roche), the PCR was carried out in a total volume of 20μL containing 5μL of extracted genomic DNA or plasmids including the gene targeted for each species as positive control. The mixture to detect *P*. *falciparum* contained 0.2 mM dNTPs, 3.5 mM MgCl_2_, 0.4 μM of forward primer, 0.5 μM of reverse primer, 0.15 μM each of the donor and acceptor probes, 0.25 g/l bovine serum albumin (BSA) and 0.5 U Taq polymerase (Platinum, Invitrogen). The multiplex PCR to detect *P*. *malariae*, *P*. *ovale* and *P*. *vivax* used three forward primers; each primer is specific of one species and 1 reverse primer for the three species. The mixture differed from the first one by primer concentration: 0.7 μM of forward primer for *P*. *malariae*, 0.5 μM of each forward primer for *P*. *ovale* and *P*. *vivax*, 0.4 μM of reverse primer.

For both PCRs, the following PCR program was used: 2 min at 95°C; 10 touchdown repeated cycles of 5 sec at 95°C, 10 sec at 63°C to 58°C, 7 sec at 72°C; and 45 repeated cycle of 5 sec at 95°C, 10 sec at 58°C, 7 sec at 72°C. Melting analysis was performed by denaturing for 30 sec at 95°C and cooling for 2 min to 45°C followed by heating at the rate of 0.1°C/s from 45°C to 75°C.

## Results

### Coastal locations

Plasma samples from the two coastal areas (Mananjary and Manakar) from 433 pregnant women were investigated. Each sample was analyzed by ELISA in two independent experiments. For 421 samples the ELISA result was negative. For the remaining 12 samples, the ratios were borderline or positive. In case of doubt, a third ELISA was performed (see Table 1 for details).

**Table 1:** Serological test results for 12 suspected anti-ZIKV seropositive from two coastal towns in Madagascar.

ID	ELISA Ratio 1 IgG (ZIKV)	ELISA Ratio 2 IgG (ZIKV)	ELISA Ratio 3 IgG (ZIKV)	Interpretation of ELISA tests ^#^ (ZIKV)	IIFA (ZIKV)	VNT (ZIKV)	P.-falciparum PCR	Anti-CHIKV-IgG	Anti-DENV-IgG
*M0042*	0.82 Borderline	0.68 Negative		Negative			Negative	Negative	Negative
*M0790*	0.84 Borderline	0.91 Borderline		Negative			Negative	Positive	Positive
*M0693*	0.87 Borderline	0.53 Negative		Negative			Negative	Negative	Negative
*M0094*	1.05 Borderline	1.08 Borderline		Negative			Negative	Negative	Negative
*M0717*	1.05 Borderline	0.54 Negative		Negative			Negative	Negative	Negative
*M0711*	1.1 Positive	0.64 Negative	0.63 Negative	Negative			Positive	Negative	Negative
*M0131*	1.16 Positive	1.04 Borderline		**Positive**	Negative		Negative	Negative	Negative
*M0159*	1.51 Positive	1.23 Positive		**Positive**	**Positive**	Negative	Positive	Negative	Negative
*M0068*	1.7 Positive	1.6 Positive		**Positive**	**Positive**	Negative	Positive	Negative	Negative
*M0183*	1.72 Positive	0.74 Negative	0.33 Negative	Negative			Positive	Negative	Negative
*M0784*	1.87 Positive	1.97 Positive		**Positive**	**Positive**	Negative	Negative	Negative	Positive
*M0764*	1.92 Positive	0.69 Negative	0.73 Negative	Negative			Negative	Negative	Negative

^#^ Decision procedure: Each single measurement was classified “Negative”, “Borderline” or “Positive” according to the extinction ratio values (<0.8 negative, ≥ 0.8 - ≤1.1 borderline, >1.1 positive). For all samples, two measurements were carried out (ratio 1 and ratio 2). If both measurements were positive, the respective sample was classified positive. If both measurements were negative or both measurements were borderline, the sample was classified negative. Combinations of positive and borderline were classified positive and combinations of negative and borderline as negative. If the measurement results were discordant (positive and negative), a third decisive measurement was carried out.

All anti-ZIKV IgM tests for the coastal locations were negative (no signal or ratio <0.8).

Among the four samples from the coastal locations with a positive anti-ZIKV IgG, two were positive for *P*. *falciparum* (50%), compared to 73 (17%) of the remaining 429 anti-ZIKV-antibody negatives samples (prevalence ratio 2.9, 95% CI 1.1–8.0). Of the other two ZIKV-positive samples, one showed a positive IgG against DENV, and one was negative for both *P*. *falciparum* and DENV.

Further testing by IIFA revealed that three of the four suspected samples, M0159, M0068 and M0784, were positive for ZIKV-specific IgG and IgM antibodies. However, when tested by VNT, no neutralizing antibodies against the African ZIKV strain MR766 were detected in the three tested sera.

### Highland locations

Plasma samples from 783 pregnant women from the four highland cities (Ifanadiana, Moramanga, Ambositra, Tsiroanomandidiy) were investigated. For 779 samples the ELISA was negative. For the remaining four samples, the ratios were borderline or positive (Table 2). In contrast to the coastal samples, two of the IgG-positive samples were also positive for IgM. Among the two samples from the highland locations with a positive anti-ZIKV IgG and IgM, two were positive for *P*. *falciparum* (100%), compared to 79 (10%) of the 781 anti-ZIKV-antibody negatives (prevalence ratio 9.9, 95% CI 8.0–12.2). The two samples that were positive both in the anti-ZIKV IgG ELISA and the *P*. *falciparum* PCR were also the only samples in the dataset that were clearly positive in the anti-ZIKV IgM ELISA. Confirmatory testing by IIFA was negative for both suspected samples.

**Table 2:** Serological test results for four suspected anti-ZIKV seropositive samples from four highland locations in Madagascar.

ID	ELISA Ratio 1 IgG (ZIKV)	ELISA Ratio 2 IgG (ZIKV)	Interpretation of ELISA tests ^#^ (ZIKV)	IIFA (ZIKV)	P. fal-ciparum PCR	Anti-CHIKV-IgG	Anti-DENV-IgG	Ratio 1 IgM (ZIKV)	Ratio 2 IgM (ZIKV)
*M0401*	1.49 Positive	1.75 Positive	**Positive**	Negative	**Positive**	Negative	Negative	1.96 Positive	1.99 Positive
*M0455*	1.54 Positive	1.2 Positive	**Positive**	Negative	**Positive**	Negative	Negative	3.02 Positive	3.54 Positive
*M0897*	0.8 Borderline	0.82 Borderline	Negative		Negative	Negative	Negative	0.03 Negative	0.03 Negative
*M0871*	0.85 Borderline	0.06 Negative	Negative		Negative	Negative	Negative		

^#^ Decision procedure: Each single measurement was classified “Negative”, “Borderline” or “Positive” according to the extinction ratio values (<0.8 negative, ≥ 0.8 - ≤1.1 borderline, >1.1 positive). For all samples, two measurements were carried out (ratio 1 and ratio 2). If both measurements were positive, the respective sample was classified positive. If both measurements were negative or both measurements were borderline, the sample was classified negative. Combinations of positive and borderline were classified positive and combinations of negative and borderline as negative. If the measurement results were discordant (positive and negative), a third decisive measurement was carried out.

## Discussion

The study results lead to two statements: First, the presence of ZIKV antibodies in pregnant women from Madagascar in 2010 is unlikely. Second, malaria parasites may interfere with anti-ZIKV ELISA tests and may lead to false positive test results.

Outbreaks of CHIKV and DENV infections, other arboviral diseases, occurred during the last 10 years in Madagascar [19, 24, 25]. In contrast, ZIKV infections have not been described clinically or serologically in Madagascar so far.

A limitation of our study is that only six of the 1,216 ZIKV IgG-ELISA tests performed in our study were positive. While this is a small number and limits statistical interpretability, we cannot conclude on the presence of ZIKV virus in the human population of Madagascar before 2010, without considering the possibility of the positive tests being “false positives”. At the coast we found four positive samples among 433. Based on our binomial assumptions, the probability of having at least four (false) positive samples among 433 is only 1.2% when assuming a specificity of 99.8%. We would conclude that at least some of our positive ELISA test results were probably true positives. However, we have to assume that malaria infection interferes with the anti-ZIKV ELISA test, so its specificity is lowered in our malaria-endemic study population. Although prevalence ratios for the association between ZIKA-test positivity and malaria infections were relatively high (10 in the highlands and 2.9 at the coast), we always have to keep in mind that they contained only two anti-ZIKV positive samples in the highlands and four at the coast.

Notably, the frequency of malaria-PCR positivity was higher in the few ZIKV-ELISA positives than for ZIKV-ELISA negatives, hence malaria may have led to (false) ZIKV-ELISA positives.

However, not all ZIKV-ELISA positive samples were also malaria positive, indeed two anti-ZIKV IgG positive samples were malaria negative. When using the specificity without considering malaria (99.8%), the probability for obtaining at least two false positives among 433 samples is 22%.

Cross-reactivity was expected towards flaviviruses that exhibit a high degree of sequence and structural homology. Especially the envelope (E) glycoprotein is very similar among flaviviruses, whereas the secreted NS1 protein is more conserved and ZIKV-specific.

Van Esbroeck *et al*. confirmed the high ZIKV specificity claimed by the EUROIMMUN assay [16], using plasma of 10 PCR-confirmed dengue patients, which all tested negative for anti-ZIKV IgGs [17]. However, in the same study it was also reported that 14 (41%) out of 34 PCR-positive malaria infections tested positive for ZIKV IgM and IgG, although ZIKV infection was excluded for 11 of these patients by VNT [17].

Three of six IIFA tests performed on ELISA-positive samples were positive for ZIKV-specific IgG and IgM antibodies, but no neutralizing antibodies against the African ZIKV strain MR766 were observed. Anti-ZIKV ELISA IgM-positive tests were only found in the two IgG-positive individuals from the highlands, both also carrying malaria parasites.

This study suggests that the association between anti-ZIKV IgG ELISA positivity and *P*. *falciparum* PCR -positivity was stronger in highland regions with a lower *P*. *falciparum* endemicity than highly endemic coastal regions.

In summary, it is important to consider the potential interference between ZIKV ELISA and *P*. *falciparum* infection as previously reported [17]. As ZIKV virus often occurs in malaria-endemic regions, this shortcoming of the ZIKV ELISA has to be taken into account.

## Supporting information

S1 File ZIKA Madagascar dataset. (XLS)
